# Recent trends in disease-modifying therapy use and associated sickness absence and disability pension among people with multiple sclerosis in Sweden

**DOI:** 10.1177/13524585231225929

**Published:** 2024-01-19

**Authors:** Fitsum Sebsibe Teni, Alejandra Machado, Katharina Fink, Hanna Gyllensten, Jan Hillert, Emilie Friberg

**Affiliations:** Division of Insurance Medicine, Department of Clinical Neuroscience, Karolinska Institutet, Stockholm, Sweden; Division of Insurance Medicine, Department of Clinical Neuroscience, Karolinska Institutet, Stockholm, Sweden; Division of Neurology, Department of Clinical Neuroscience, Karolinska Institutet, Stockholm, Sweden; Institute of Health and Care Sciences, Sahlgrenska Academy, University of Gothenburg, Gothenburg, Sweden; Division of Neurology, Department of Clinical Neuroscience, Karolinska Institutet, Stockholm, Sweden; Division of Insurance Medicine, Department of Clinical Neuroscience, Karolinska Institutet, Stockholm, Sweden

**Keywords:** Disease-modifying drugs, high-efficacy DMTs, sick leave, cluster analysis

## Abstract

**Background:**

Disease-modifying therapies (DMTs) have led to improved health and work productivity among people with multiple sclerosis (PwMS).

**Objectives:**

To describe trajectories of recent DMT use and their association with sickness absence and/or disability pension (SADP) among PwMS in Sweden.

**Methods:**

A longitudinal register–based study was conducted among 1395 PwMS with treatment start in 2014/2015. While DMT use over 5 years was assessed using sequence analysis resulting in four clusters, a 7-year (Y_−2_ toY_4_) trend of SADP was analyzed using zero-inflated negative binomial regression.

**Results:**

Four clusters of DMT use trajectories were identified: *long-term non-high-efficacy* (483, 34.6%), *long-term high-efficacy* (572, 41%), *escalation* (221, 15.8%), and *discontinuation* (119, 8.5%). Progressive MS and higher expanded disability status scale scores were associated with the *escalation, long-term high-efficacy*, or *discontinuation* clusters. PwMS in the *long-term high-efficacy* and *escalation* clusters had higher likelihood of being on SADP. However, PwMS initiating high-efficacy DMTs demonstrated steeper decline in SADP than others.

**Conclusion:**

Using sequence analysis, this study showed recent DMT use trajectories among PwMS where initiation of high-efficacy DMTs has become more common. The trend of SADP was stable and lower in those using non-high-efficacy DMTs and larger improvements were shown in those initiating high-efficacy DMTs.

## Introduction

Multiple sclerosis (MS) is a demyelinating disease of the central nervous system characterized by inflammation and neurodegeneration.^1^ It occurs more commonly among working-aged individuals.^2^ It has negative work impacts such as unemployment, reduced working hours, and loss of productivity.^3^ Increased sickness absence and/or disability pension (SADP) have also been reported.^4^

Despite no cure, increasingly effective treatments, disease-modifying therapies (DMTs) have become available.^5^ These include injectable DMTs - interferons, and glatiramer acetate - approved since the early 1990s.^6^ Another group includes the oral DMTs dimethyl fumarate, teriflunomide, and fingolimod.^7^ Monoclonal antibodies include high-efficacy infusion therapies such as the anti-CD20s, rituximab (off-label), ocrelizumab, and ofatumumab.^8^ Other monoclonal antibodies used include alemtuzumab (2013) and natalizumab (2006).^9^

According to recent studies initiating high-efficacy DMTs, induction, has become an increasingly preferred approach than escalation from low to moderate or high-efficacy DMTs.^10,11^ The positive impact of DMTs on work productivity has also been highlighted in decreased rate of early retirement,^12^ improved work productivity,^13^ and improved work ability and reduced SADP among people with multiple sclerosis (PwMS).^14^ A recent systematic review also showed an overall decrease in the prevalence of unemployment among PwMS, attributed partly to the use of DMTs which was more pronounced since 2010, when the number of high-efficacy DMTs started to increase.^15^

There is limited literature on the long-term DMT use trends and corresponding work disability measured with SADP days. In a recent register-based study, we assessed the trajectories of DMT use over 10 years from MS onset, using sequence analysis.^16^ This method provides an overall illustration of sequences of categorical states over time and a way of clustering the sequences into different groups.^17^ As we used the onset years 2007–2010 in the earlier study to assess long-term trends in DMT use, it did not capture more recent patterns of treatment initiations with already available and newer DMTs. The present study focused on recent cohorts and the time of treatment start. Hence, the objective was to describe the trajectories of DMT use in recent years and the demographic and clinical factors associated with them as well as the association of cluster membership with SADP among PwMS in Sweden.

## Methods

### Study design and period

A longitudinal register–based study was conducted among PwMS in the Swedish Multiple Sclerosis Registry (SMSreg)^18^ who started their treatment in 2014/2015. DMT use was assessed in a 5-year period, from year of first treatment start/decision (Y_0_) to 4 years after (Y_4_). Associated trends of SADP were analyzed from 2 years prior to the year of treatment start up to 4 years after (Y_−2_ to Y_4_).

### Study population

Of the 1741 PwMS in the SMSreg with first treatment start/decision in 2014/2015, 1395 were included in the analysis leaving out PwMS beyond 18–60 years (*n* = 100), with treatment start date before diagnosis (*n* = 64), and no record in population register (*n* = 134) (Supplemental Figure S1).

### Variables

The variables in the study include sociodemographic (age, sex, birth country, family composition, living area, education), clinical (MS type, Expanded Disability Status Scale (EDSS), frequency of DMT switch), and health-related quality of life (HRQoL).

### Data sources

Demographic, clinical, and HRQoL data from the SMSreg were linked to five national registers. Sociodemographic data were obtained from Longitudinal Integrated Database for Health Insurance and Labor Market Studies (LISA),^19^ and data on death during the follow-up were determined using the Swedish Cause of Death Register.^20^ Comorbidity was identified using data from the Swedish Prescribed Drug Register (SPDR)^21^ and the Swedish Cancer Register (SCR).^22^ Micro-Data for Analysis of the Social Insurance System (MiDAS)^23^ was used to determine SADP days.

### States of DMT use

PwMS used a total of 14 DMTs during the follow-up which were categorized into high-efficacy and non-high-efficacy DMTs, based on their effect on relapse and disability progression according to systematic reviews and consultation with neurologists, as described in our previous study and presented in Supplemental Table S1.^16^ Hence, DMT use was categorized into three states: high-efficacy, non-high-efficacy, and no DMTs.

### SADP

In this study, net SADP days (calculated using gross days and extent/percentage of SADP) were determined using data from MiDAS on a yearly basis starting from 2 years (Y_−2_) preceding first treatment start/decision year up to 4 years after (Y_4_) (Supplementary Material).

### Comorbidity

Comorbidity was determined using Rx-Risk index, which identifies comorbidity through prescribed drugs. A record of drugs besides DMTs prescribed and dispensed to PwMS during the year of DMT start was identified from SPDR.^21^ In addition, data on cancer diagnosis before and during the treatment start year, from SCR, were included in the comorbidity assessment.^22^

### HRQoL

Data on the EuroQol five-dimension questionnaire (EQ-5D) from the SMSreg was used to measure HRQoL. The closest EQ-5D-3L (three-level version) data within 2 years before or after the first treatment start date were used. EQ-5D index generally ranges from zero (*dead*) to one (*full health*). Negative (worse than dead) EQ-5D index values are also parts of a number of value sets. We used the Swedish experience-based EQ-5D-3L value set—where the resulting index ranges from 0.34 to 0.97—to calculate HRQoL.^24^

### Statistical analyses

Descriptive analysis was used to summarize distribution of PwMS between the two treatment start years, 2014 and 2015. Accordingly, sociodemographic, clinical, HRQoL, and SADP data were compared. Chi-square, independent *t*-tests, and Mann–Whitney *U*-tests were used to assess the significance of distribution of the above variables by treatment start year.

Trajectories of DMT use over the follow-up were assessed using sequence analysis which was performed in three main steps. First, a sequence object—a sequence of DMT use states for each of the PwMS during the follow-up—was prepared. The DMT use states were determined for every 3-month period from the date of first treatment start (Y_0_) up to 5 years after (Y_4_). Second, the dissimilarities across the DMT use sequences of PwMS were determined using a common dissimilarity measure, optimal matching.^25^ Third, the information from the sequences and the dissimilarity measures were used to group the sequences into clusters. Clustering was performed using a combination of hierarchical clustering and partitioning around medoid (PAM) approaches.^26^ Two to 12 clusters were assessed to choose those with the highest quality per statistical criteria (e.g. Average Silhouette’s Width (ASW)) and clinical plausibility.^26^ Accordingly, DMT use trajectories with three and four clusters were the ones with the highest and second highest ASW values, respectively (Supplemental Table S2). However, presence of the clinically common trend of escalation from non-high-efficacy to a high-efficacy DMT led to choosing four clusters of DMT use trajectories.

The association of different demographic, clinical and HRQoL variables with cluster membership of PwMS was assessed using multinomial logistic regression analysis. The association of cluster membership with SADP days was assessed using zero-inflated negative binomial (ZINB) regression analysis.^27^ It is suited to handle distributions with large proportions of zeros as in the SADP data in this study. Accodingly, ZINB regression has two components: binary logistic regression (occurrence of SADP vs no SADP days) and negative binomial regression (the number of SADP days).

R version 4.1.2 (R Foundation for Statistical Computing, Vienna, Austria) and SAS version 9.4 (SAS Institute Inc., Cary, NC, USA) were used in the analysis. A *p*-value of 0.05 was used as a cut-off point for statistical significance.

### Results

Demographic, clinical, and HRQoL data of the PwMS shown in Table 1. Overall, they were comparable by year of treatment start, while the 2014 cohort had more treatment changes. Two years prior to treatment start, significantly lower SADP was noted in the 2014 cohort (Supplemental Table S3).

**Table 1.** Demographic and clinical characteristics of PwMS across the four DMT use clusters.

Variable	Cluster				Total	*p*
	Long-term non-high-efficacy DMTs	Long-term high-efficacy DMTs	Escalation to high-efficacy DMTs	Discontinued/no DMTs		
	*n* = 483	*n* = 572	*n* = 221	*n* = 119	*n* = 1395	
	% (n)	% (n)	% (n)	% (n)	% (n)	
Sex						**0.0158**
Women	70.6 (341)	65.0 (372)	71.9 (159)	78.2 (93)	69.2 (965)	
Men	29.4 (142)	35.0 (200)	28.1 (62)	21.8 (26)	30.8 (430)	
Age groups (years)						**0.0003**
19–25	10.1 (49)	14.5 (83)	14.9 (33)	5.0 (6)	12.3 (171)	
26–35	29.6 (143)	29.7 (170)	28.5 (63)	24.4 (29)	29.0 (405)	
36–45	32.9 (159)	23.6 (135)	31.2 (69)	26.9 (32)	28.3 (395)	
46–55	20.9 (101)	25.2 (144)	21.7 (48)	30.3 (36)	23.6 (329)	
56–60	6.4 (31)	7.0 (40)	3.6 (8)	13.4 (16)	6.8 (95)	
Age (mean (SD))	38.8 (9.9)	38.6 (11.1)	37.8 (10.3)	42.8 (10.3)	38.9 (10.6)	**0.0003** ^a^
Birth country						
Sweden	88.2 (426)	88.5 (506)	87.8 (194)	85.7 (102)	88.0 (1228)	0.8655
Other	11.8 (57)	11.5 (66)	12.2 (27)	14.3 (17)	12.0 (167)	
Family composition						0.0626
Married/cohabitant without children < 18 years	13.0 (63)	16.4 (94)	10.0 (22)	16.8 (20)	14.3 (199)	
Married/cohabitant with children	42.2 (204)	33.0 (189)	39.4 (87)	40.3 (48)	37.8 (528)	
Single without children	37.9 (183)	43.5 (249)	41.6 (92)	35.3 (42)	40.6 (566)	
Single with children	6.8 (33)	7.0 (40)	9.0 (20)	7.6 (9)	7.3 (102)	
Type of living area						**0.0495**
Big cities	36.9 (178)	44.1 (252)	36.7 (81)	38.7 (46)	39.9 (557)	
Medium-sized cities	42.4 (205)	38.8 (222)	47.5 (105)	37.0 (44)	41.3 (576)	
Rural areas	20.7 (100)	17.1 (98)	15.8 (35)	24.4 (29)	18.8 (262)	
Educational level^b^						0.9237
Elementary school (0–9 years)	9.9 (48)	11.7 (67)	10.0 (22)	10.9 (13)	10.8 (150)	
High school (10–12 years)	44.7 (216)	46.9 (268)	46.6 (103)	43.7 (52)	45.8 (639)	
University/college (>12 years)	45.1 (218)	40.9 (234)	43.0 (95)	45.4 (54)	43.1 (601)	
Type of multiple sclerosis^c^						**<0.0001**
Relapsing-remitting	95.4 (461)	79.7 (456)	92.3 (204)	61.3 (73)	85.6 (1194)	
Progressive (primary + secondary)^d^	4.1 (20)	19.4 (111)	7.7 (17)	37.0 (44)	13.8 (192)	
EDSS score (closest to treatment start)^e^						**<0.0001**
0–2.5	74.9 (362)	63.1 (361)	67.9 (150)	50.4 (60)	66.9 (933)	
3–8.5	8.9 (43)	28.3 (162)	16.7 (37)	34.5 (41)	20.3 (283)	
Missing	16.1 (78)	8.6 (49)	15.4 (34)	15.1 (18)	12.8 (179)	
Comorbidity index						**0.0163**
0	11.8 (57)	9.1 (52)	6.3 (14)	8.4 (10)	9.5 (133)	
1–2	51.1 (247)	47.9 (274)	51.6 (114)	48.7 (58)	49.7 (693)	
3–4	24.0 (116)	26.9 (154)	32.6 (72)	21.8 (26)	26.4 (368)	
5+	13.0 (63)	16.1 (92)	9.5 (21)	21.0 (25)	14.4 (201)	
Frequency of DMT switch during follow-up						**<0.0001**
0	51.6 (249)	60.0 (343)	0.0 (0)	56.3 (67)	47.2 (659)	
1	33.7 (163)	29.4 (168)	62.4 (138)	24.4 (29)	35.7 (498)	
2	9.7 (47)	8.9 (51)	25.8 (57)	5.9 (7)	11.6 (162)	
3+	5.0 (24)	1.7 (10)	11.8 (26)	5.0 (6)	4.7 (66)	
No DMT	0.0 (0)	0.0 (0)	0.0 (0)	8.4 (10)	0.7 (10)	
EQ-5D index (mean (SD))^f^	0.86 (0.12)	0.82 (0.13)	0.83 (0.13)	0.81 (0.14)	0.84 (0.13)	**<0.0001** ^a^
EQ VAS score (mean (SD))^f^	72.2 (21.4)	66.7 (21.3)	69.2 (21.1)	60.1 (25.4)	68.6 (21.8)	**<0.0001** ^a^
Time from MS onset to treatment start/decision (in months) (median (IQR))	12.8 (47.3)	12.9 (48.8)	12.0 (40.7)	51.4 (151.0)	14.3 (55.3)	**<0.0001** ^g^
MS treatment start year						**0.0005**
2014	54.9 (265)	44.2 (253)	57.9 (128)	52.1 (62)	50.8 (708)	
2015	45.1 (218)	55.8 (319)	42.1 (93)	47.9 (57)	49.2 (687)	

DMT: disease-modifying therapy; EDSS: Expanded Disability Status Scale—this scale measures level of disability from a score 0 (no disability) to 10 (dead); EQ-5D: EuroQol five-dimension questionnaire; EQ VAS: visual analogue scale in the EQ-5D; IQR: interquartile range; PwMS: people with multiple sclerosis; SD: standard deviation.

Statistically significant results at 0.05 are shown in bold.

a One-way analysis of variance test.

b Missing observations (*n* = 5).

c Missing observations (*n* = 9).

d Primary progressive (*n* = 80) and secondary progressive (*n* = 112).

e EDSS: 3–5.5 (*n* = 231) and EDSS: 6–8.5 (*n* = 52).

f Within 2 years before/after treatment start.

g Kruskal–Wallis test before/after treatment start.

### DMT use trajectories

**Figure 1.**
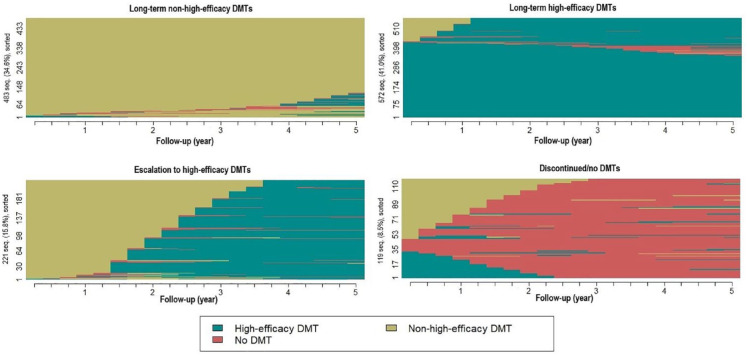
Sequence index plot of the four disease-modifying therapy (DMT) use clusters showing the states people with multiple sclerosis were in during the follow-up.

The total number of distinct sequences was 233 and the 10 most frequent ones were observed among 65.9% of the PwMS (Supplemental Figure S2). Four clusters of DMT use trajectories were chosen owing to high cluster quality and their depiction of clinical practices (cluster names are presented in *italics*) (Figure 1 and Supplemental Table S2).

Most PwMS were grouped in the *long-term high-efficacy DMTs* cluster (41.0%; hereafter *long-term high-efficacy*). Around one-third were in the *long-term non-high-efficacy DMTs* cluster (34.6%; hereafter *long-term non-high-efficacy*). The remaining PwMS were categorized in the *escalation to high-efficacy DMTs* (15.8%; hereafter *escalation*) and *discontinued/no DMTs* (8.5%; hereafter *discontinuation*) clusters (Figure 1).

The distribution of demographic, clinical, and HRQoL across DMT use trajectories showed statistically significant differences in most of the variables except birth country, family composition, and education (Table 1).

Mutually adjusted multinomial logistic regression showed variations in cluster membership by several variables. While the youngest PwMS (19–25 years) were more likely (odds ratio (OR) = 1.73 (95% confidence interval (CI) = 1.12–2.67)) to be in the *long-term high-efficacy* cluster than those aged 26–35 years, it was less likely among older PwMS (36–45 and 56–60 years). Primary and secondary progressive MS were associated with the clusters *long-term high-efficacy* and *discontinuation* and all the other three clusters, respectively (Table 2).

**Table 2.** Mutually adjusted multinomial logistic regression model of factors associated with membership to the different clusters of disease-modifying therapy use (*n* = 1376).

Variable	Cluster			
	Long-term non-high-efficacy DMTs (*n* = 481)	Long-term high-efficacy DMTs (*n* = 567)	Escalation to high-efficacy DMTs (*n* = 221)	Discontinued/no DTs (*n* = 107)
	AOR [95% CI]	AOR [95% CI]	AOR [95% CI]	AOR [95% CI]
Sex				
Men	1.00	1.22 [0.92–1.62]	1.01 [0.69–1.49]	0.60 [0.35–1.02]
Age group (years)				
19–25	1.00	**1.73 [1.12–2.67]**	1.28 [0.71–2.31]	0.53 [0.19–1.48]
36–45	1.00	**0.63 [0.45–0.89]**	1.14 [0.73–1.78]	0.70 [0.39–1.26]
46–55	1.00	0.72 [0.50–1.05]	1.25 [0.75–2.07]	0.72 [0.38–1.36]
56–60	1.00	**0.39 [0.21–0.74]**	0.65 [0.26–1.63]	0.50 [0.20–1.26]
Type of multiple sclerosis				
Primary progressive	1.00	**7.99 [3.21–19.92]**	1.38 [0.31–6.14]	**12.74 [4.36–37.23]**
Secondary progressive	1.00	**3.05 [1.58–5.86]**	**2.44 [1.04–5.74]**	**7.32 [3.28–16.38]**
Comorbidity				
1–2	1.00	1.29 [0.82–2.01]	1.33 [0.68–2.58]	1.18 [0.53–2.63]
3–4	1.00	1.45 [0.89–2.36]	1.73 [0.86–3.47]	0.98 [0.41–2.34]
5+	1.00	1.59 [0.92–2.75]	0.64 [0.28–1.47]	1.29 [0.51–3.24]
EDSS				
3–5.5	1.00	**2.24 [1.48–3.40]**	**1.92 [1.11–3.33]**	**2.15 [1.12–4.11]**
6–8.5	1.00	**7.62 [1.71–34.02]**	4.56 [0.68–30.61]	**8.90 [1.74–45.58]**
Frequency of DMT switch	1.00	**0.78 [0.65–0.93]**	**2.67 [2.18–3.26]**	1.04 [0.58–2.02]
EQ-5D index				
Lower than median	1.00	**1.54 [1.12–2.13]**	1.45 [0.96–2.19]	1.40 [0.78–2.49]
MS treatment start year				
2015	1.00	**1.57 [1.21–2.04]**	1.05 [0.74–1.48]	1.14 [0.74–1.78]

DMT: disease-modifying therapy, EDSS: expanded disability status scale, AOR: adjusted odds ratio.

Reference groups: women; 26–35 years; relapsing-remitting; 0 comorbidity; EDSS 0–2.5; EQ-5D index higher than median; MS treatment start in 2014.

Statistically significant results are shown in bold.

Similarly, PwMS with EDSS score of 3+ were more likely to be in the other clusters than the *long-term non-high-efficacy* one. The frequency of DMT switches was associated with being in the escalation cluster but had lower odds of being in the *long-term high-efficacy* cluster. However, lower HRQoL at treatment start was associated with being in the *long-term high-efficacy* cluster. The 2015 cohort were more likely to be in the *long-term high-efficacy* cluster (Table 2).

### SADP across DMT use trajectories

**Figure 2.**
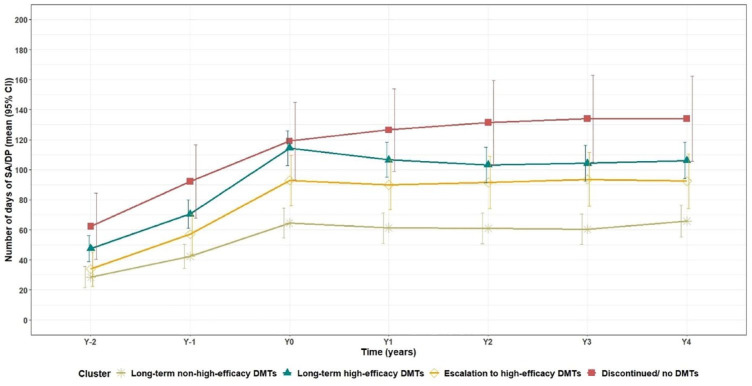
Mean sickness absence and/or disability pension days per year among the people with multiple sclerosis across disease-modifying therapy use clusters. SADP: sickness absence and/or disability pension, CI: confidence interval.

Since 2 years before treatment start, mean SADP increased with steeper increases among PwMS in clusters other than *long-term non-high-efficacy*. Since treatment start, PwMS in the *long-term high-efficacy* cluster had steeper decline than the others for 3 years followed by a stable trend. Throughout the follow-up, PwMS in the *long-term non-high-efficacy* cluster had significantly lower mean SADP days than those in the *long-term high-efficacy* and *discontinuation* clusters (Figure 2).

**Figure 3.**
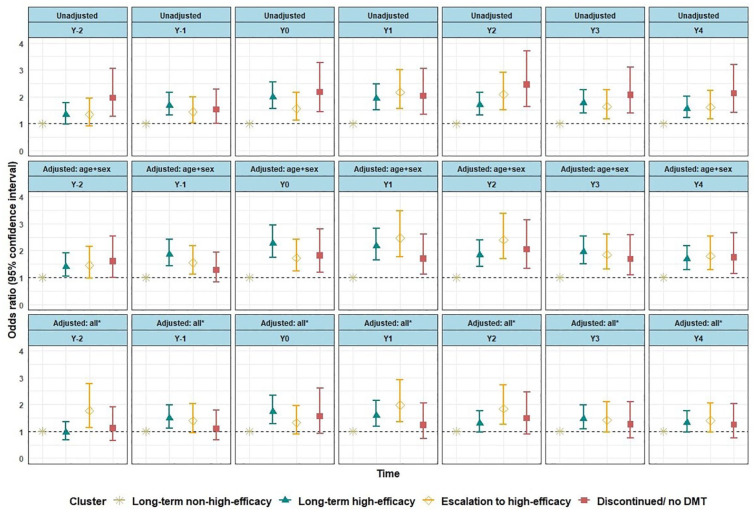
Binary logistic regression component of the zero-inflated negative binomial regression analysis on the odds of occurrence of SADP days. DMT: disease-modifying therapy, EDSS: Expanded Disability Status Scale, SADP: Sickness Absence and/or Disability Pension. *All: adjusted for the variables sex, age, type of MS, comorbidity, EDSS score, DMT switch, EQ-5D index, and MS treatment start year; reference group: long-term non-high-efficacy DMTs cluster. The models in the figure present results of the binary logistic regression component of the zero-inflated negative binomial regression, where the odds of occurrence of SADP days were compared across DMT use clusters in each year of the total 7 years of follow-up.

The binary logistic regression component of the ZINB model, adjusted for demographic, clinical, HRQoL, and treatment start year variables, showed that in the year before and 2 years since treatment start, PwMS in the *long-term high-efficacy* cluster still had significantly higher odds of being on SADP. However, in 2 of the last 3 years (Y_2_ and Y_4_), it had no significant differences compared to PwMS on non-high-efficacy DMTs (Figure 3).

**Figure 4.**
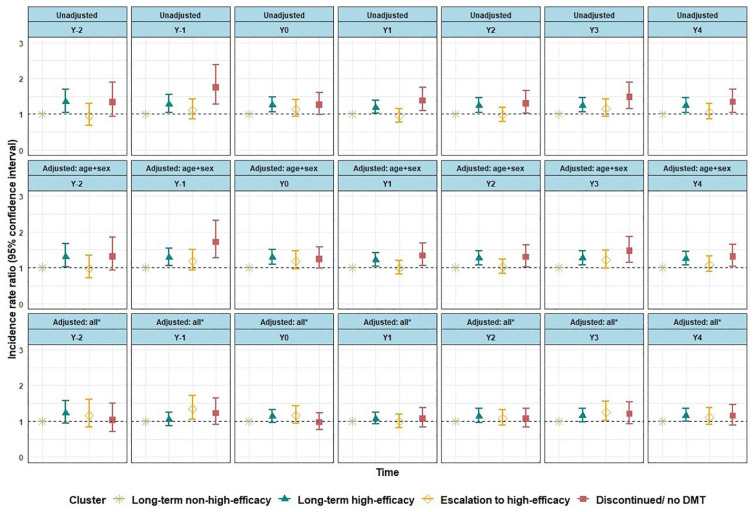
Negative binomial model component of the zero-inflated negative binomial regression analysis on the number of SADP days. DMT: disease-modifying therapy, EDSS: Expanded Disability Status Scale, SADP: Sickness Absence and/or Disability Pension. *All: adjusted for the variables sex, age, type of MS, comorbidity, EDSS score, DMT switch, EQ-5D index, and MS treatment start year; reference group: long-term non-high-efficacy DMTs cluster. The models in the figure show results of negative binomial regression component of the zero-inflated negative binomial regression, where the number of SADP days was compared across DMT use clusters in each year of the total 7 years of follow-up.

In the negative binomial model component, the final adjusted model, only PwMS in the *escalation* cluster had a significantly higher number of SADP days than those in the *long-term non-high-efficacy* cluster in the year prior to treatment start and in the fourth year (Y_3_) on treatment (Figure 4).

## Discussion

This study found four clusters of DMT use trajectories over the 5 years from DMT start: *long-term non-high-efficacy* (34.6%), *long-term high-efficacy* (41%), *escalation* (15.8%), and *discontinuation* (8.5%). Age, type of MS, EDSS score, frequency of DMT switch, and HRQoL were associated with DMT use clusters. PwMS in the *long-term high-efficacy* and *escalation* clusters had higher odds of being on SADP during portions of the follow-up.

In a previous study, we assessed long-term trends in DMT use and corresponding trajectories of SADP. It included PwMS with onset during 2007–2010.^16^ This study, however, included PwMS with recent treatment initiation while making a long-term observation. The four clusters identified were largely comparable with the findings from our earlier study—three of them were similar in the two studies. One cluster showed a different pattern. That is, a *long-term high-efficacy* cluster was identified in this study while *delayed start and escalation to high-efficacy* DMTs was observed in the previous one.^16^ The difference in the starting point of observation and the increased early initiation of high-efficacy DMTs, together with their increased availability, in the recent cohorts could explain the *long-term high-efficacy* cluster.^11,28^ In comparison to our previous study, the increased use of high-efficacy DMTs observed in this study was demonstrated by the higher proportion of PwMS who had no or one DMT switch—from two-thirds in the previous study to more than 80% in this study. Furthermore, the increased use of high-efficacy DMTs more recently relates to the increased availability and use of high-efficacy DMTs in Sweden, particularly the highly increased use of rituximab in the treatment of MS since early 2010s as well as the availability of other high-efficacy DMTs in late 2000s and 2010s including natalizumab, alemtuzumab, ocrelizumab, and others.^16,28^

Our findings had common features also with a study from France which reported four clusters where the *first-line DMTs* cluster roughly compares with the *long-term non-high-efficacy* cluster in our study. The *second-line DMTs* cluster had similarities with the *long-term high-efficacy* and *escalation* clusters in our study. The *not treated* cluster was comparable with the *discontinuation* cluster in our study.^29^ However, unlike in the French study (*first-line DMTs*), the *long-term high-efficacy* cluster was the most common in our study which could partly be due to the earlier PwMS cohort (2010) in the cited study.^29^

The significantly lower odds of being in the *long-term high-efficacy* cluster in older ages in our study were comparable with the French study in belonging to the *second-line DMTs* cluster.^29^ Older PwMS had lower odds of belonging to the *escalation* cluster also in our previous study, but there was no such association in this study.^16^ This could be because early use of high-efficacy DMTs was lower in the previous study which has increased more recently and such sequences were previously grouped mainly as part of the *escalation* cluster.^16^ The decreasing benefits of high-efficacy DMTs over non-high-efficacy ones among older PwMS have been highlighted in the literature.^30^

Progressive forms of MS were associated with escalating to (secondary progressive), being on high-efficacy DMTs, or discontinuing/not being on DMT similar to our previous study.^16^ The pattern reflects the limited DMT options to treat progressive MS, potentially associated with the relatively limited understanding of their pathology than relapsing MS.^31^ Specific DMTs approved for progressive MS are ocrelizumab for primary and siponimod for secondary progressive MS.^32^

Higher EDSS scores around treatment start were also associated with belonging in the DMT use clusters other than *long-term non-high-efficacy*, similar to our previous study.^16^ The higher odds of initiating high-efficacy DMTs are seemingly in line with the increasingly common induction approach.^10,11,28^ Similarly, the higher odds of escalation among PwMS with higher EDSS score also show switch to high-efficacy DMTs among those who initiated non-high-efficacy DMTs and have not had the desired effect. A similar association of higher initial EDSS score with escalation has been reported previously.^33^ Furthermore, the higher odds of discontinuation/not taking DMTs could indicate severe disease where DMTs had not been effective. The French study also found moderate to severe disability to be associated with being in the *not treated* cluster.^29^ In addition, this cluster also contains a small proportion of individuals who did not take DMTs throughout follow-up, which were generally at worse MS status than other PwMS in the cluster.

Lower HRQoL around treatment start was associated with initiating high-efficacy DMTs which was similar to the above finding that PwMS with severe disease were more likely to initiate high-efficacy DMTs. The finding shows HRQoL aspect of worse health among PwMS which might have led to initiating higher efficacy DMTs.

The 7-year trends in SADP days across the different DMT use clusters showed that individuals in the *long-term non-high-efficacy* cluster had lower SADP days than PwMS in the *long-term high-efficacy* and *discontinuation* clusters. This trend was similar to our findings in the previous study.^16^ In both studies, we observed that a significant proportion of PwMS who were in the *long-term non-high-efficacy* cluster were relatively healthier—lower EDSS scores, and in this study, better HRQoL around treatment start.^16^ This could explain the relatively lower SADP days in this cluster throughout the follow-up. The lower SADP days were also evident in the findings of the negative binomial (unadjusted and age–sex-adjusted) section of the ZINB regression, although this did not remain in the models adjusted for demographic, clinical, HRQoL, and treatment start year variables. Lower risk of loss of income and DP among PwMS with stable disease was reported by a study from Denmark.^34^

In the years prior to treatment start, although SADP days of all PwMS increased, it was higher in the *discontinuation* cluster than in the *long-term non-high-efficacy* cluster which could relate to the relatively worse baseline health in this group leading to more SADP days. This was in line with our findings in the previous study.^16^

PwMS in the *long-term high-efficacy* cluster showed a relatively steeper decline in mean SADP days compared to the other clusters in the 2 years since treatment start and remained stable after that. This could partly explain the larger improvement among PwMS who initiated high-efficacy DMTs from treatment start. In addition, the lower likelihood of DMT switch in this cluster could have also contributed to this finding. Furthermore, their worse baseline health status than PwMS in the *long-term non-high-efficacy* and *escalation* clusters could also indicate the need for high-efficacy DMTs and the subsequent larger improvement.

Despite the larger improvement among PwMS in the *long-term high-efficacy* cluster, they had higher odds of being on SADP than those in the *long-term non-high-efficacy* cluster which remained in many of the follow-up years. This, as discussed above, could reflect more severe disease among a section of the PwMS which led to treatment with high-efficacy DMTs. On the contrary, PwMS in the *escalation* cluster showed higher odds of SADP in the second and third year since treatment start. This trend could be indicative of the time when lack of/limited improvement of MS led to escalation from non-high-efficacy to high-efficacy DMTs reflected in the higher occurrence of SADP. Similar to DMT use cluster membership, having progressive forms of MS has been generally associated with higher odds of SADP in this study.

This study has several strengths including the use of sequence analysis which has helped in presenting the trends in DMT use, in real-world clinical practice, in a visually illustrative and comprehensible manner. In addition, the study showed long-term DMT use trajectories among recent cohorts of PwMS revealing the current shift to treatment initiation with high-efficacy DMTs. Furthermore, the use of data linked across several nationwide registers allowed for the consideration of many variables in the analyses. The study also had limitations which need to be considered in interpreting the findings. EDSS scores closest to the date of treatment start were employed to reduce missing observations if otherwise only observations at treatment start were included. Similarly, HRQoL data within 2 years before/after treatment start date was used to minimize missing observations. Furthermore, another limitation is that this study does not provide data on the type of job activity which would show the distribution of PwMS in different areas of work in relation to disease course and treatment. In addition, specific conditions at work such as possibilities for adaptation^35^ and work barriers^36^ could be important for an individual’s possibilities to continue working. While the use of sequence analysis provided an illustrative approach to describe DMT use trends, its inability to accommodate the impact of time-varying variable on clusters is a notable limitation.

This study adds important information to the literature on the pattern of long-term DMT use and the associated SADP trends. The trends reflect the disease course which led to specific DMT use trajectories and the impact DMTs may have had over time. The findings also support the evidence on recent change in treatment strategy to early initiation of high-efficacy DMTs which could coincide with earlier diagnosis. This indicates SADP could be useful measures to assess implications of MS disease course and DMTs on the health and ability to work among PwMS. Further studies comparing high- and non-high-efficacy DMTs in terms of their impact on SADP could be useful to measure the implications of different treatments.

## Conclusion

Through sequence analysis, this study showed recent DMT use trajectories among PwMS with the initiation of high-efficacy DMTs becoming more common. Several demographic and clinical variables and HRQoL were found to be associated with DMT use trajectories. Lower levels of SADP were noted among PwMS on long-term non-high-efficacy DMTs while those initiating or escalating to long-term high-efficacy DMTs were more likely to be on SADP. Despite this, PwMS initiating long-term high-efficacy showed the largest decrease in SADP.

## Supplemental Material

sj-pdf-1-msj-10.1177_13524585231225929 – Supplemental material for Recent trends in disease-modifying therapy use and associated sickness absence and disability pension among people with multiple sclerosis in Sweden Supplemental material, sj-pdf-1-msj-10.1177_13524585231225929 for Recent trends in disease-modifying therapy use and associated sickness absence and disability pension among people with multiple sclerosis in Sweden by Fitsum Sebsibe Teni, Alejandra Machado, Katharina Fink, Hanna Gyllensten, Jan Hillert and Emilie Friberg in Multiple Sclerosis Journal
