# Supplementary material for: Recent trends in disease-modifying therapy use and associated sickness absence and disability pension among people with multiple sclerosis in Sweden
Source: Mult Scler. 2024 Jan 19;30(3):419–31. doi: 10.1177/13524585231225929 (PMC10935615; doi:10.1177/13524585231225929)
Supplement: sj-pdf-1-msj-10.1177_13524585231225929 – Supplemental material for Recent trends in disease-modifying therapy use and associated sickness absence and disability pension among people with multiple sclerosis in Sweden [file sj-pdf-1-msj-10.1177_13524585231225929.pdf]

Supplementary material

Recent trends in disease-modifying therapy use and associated sickness absence and disability pension among people with multiple sclerosis in Sweden

Authors

Fitsum Sebsibe Teni[1*], Alejandra Machado[1], Katharina Fink[2], Hanna Gyllensten[3], Jan Hillert[2], Emilie Friberg[1]

[1]Division of Insurance Medicine, Department of Clinical Neuroscience, Karolinska Institutet, SE-171 77 Stockholm, Sweden

[2]Division of Neurology, Department of Clinical Neuroscience, Karolinska Institutet, SE-171 77 Stockholm, Sweden

[3]Institute of Health and Care Sciences, Sahlgrenska Academy, University of Gothenburg, SE-405 30, Gothenburg, Sweden

*Correspondence: Fitsum Sebsibe Teni, fitsum.teni@ki.se

Content

Methodology 2

Table S1. Classification of disease-modifying therapies into high and non-high-efficacy categories 3

Figure S1. Selection of people with multiple sclerosis included in the study [LISA: Longitudinal Integrated Database for Health Insurance and Labor Market Studies; PwMS: people with multiple sclerosis; MS: multiple sclerosis] [An individual might fulfil more than one exclusion criteria and the number of PwMS could appear more] 4

Table S2. Quality assessment metrics for the clusters of disease-modifying therapy use trajectories 5

Table S3. Descriptive statistics on sociodemographic, clinical, health-related quality of life and sickness absence and disability pension data of PwMS by treatment start year (n=1395) 6

Figure S2. The ten most common sequences of disease-modifying therapies among the people with multiple sclerosis [The sequences of DMT use states shown here present the ten most frequent ones which account for about two-thirds (65.9%) of the total 1395. The total number of sequences (1 for each of the PwMS) are categorized in to the clusters as shown in the findings in the main text of the manuscript based on their similarity/dissimilarity. DMT: disease-modifying therapy] 7

References 8

Methodology

Sickness absence and disability pension

In this study, the data on SA included spells longer than 14 days to prevent bias in relation to unemployed individuals. Meanwhile for employed individuals, employers pay from the 2nd to the 14th day of SA, eligible unemployed individuals receive benefits from the SIA from the 2nd day.

In Sweden, individuals aged 16+ years with income (a minimum of 24% of the annual price base amount) from work or unemployment benefits are eligible to SA benefits from the Social Insurance Agency (SIA). Furthermore, individuals aged 19-64 years with long-term/permanently reduced work capacity, despite the presence of previous income from work or not, are eligible to receive DP. Both SA and DP can be provided on a full or part-time basis (25%, 50% or 75%). SA and DP are paid out to cover about 80% and 64% of lost income, respectively, up to a certain level [1].

Comorbidity

Rx-Risk index identifies comorbidity through prescribed drugs. It was shown to be valid in predicting mortality and was also compared with another comorbidity measure [2,3]. It has also been used in MS previously [4].

Table S1. Classification of disease-modifying therapies into high and non-high-efficacy categories

DMT	Category
Alemtuzumab	High-efficacy DMTs
Hematopoietic stem cell transplantation (HSCT)	
Natalizumab	
Ocrelizumab	
Ofatumumab	
Rituximab	
Cladribine	Non-high-efficacy DMTs
Dimethyl fumarate	
Fingolimod	
Glatiramer acetate	
Interferons (interferon beta-1a, interferon beta-1b, and peginterferon beta-1a)	
Teriflunomide	

DMT: disease-modifying therapy

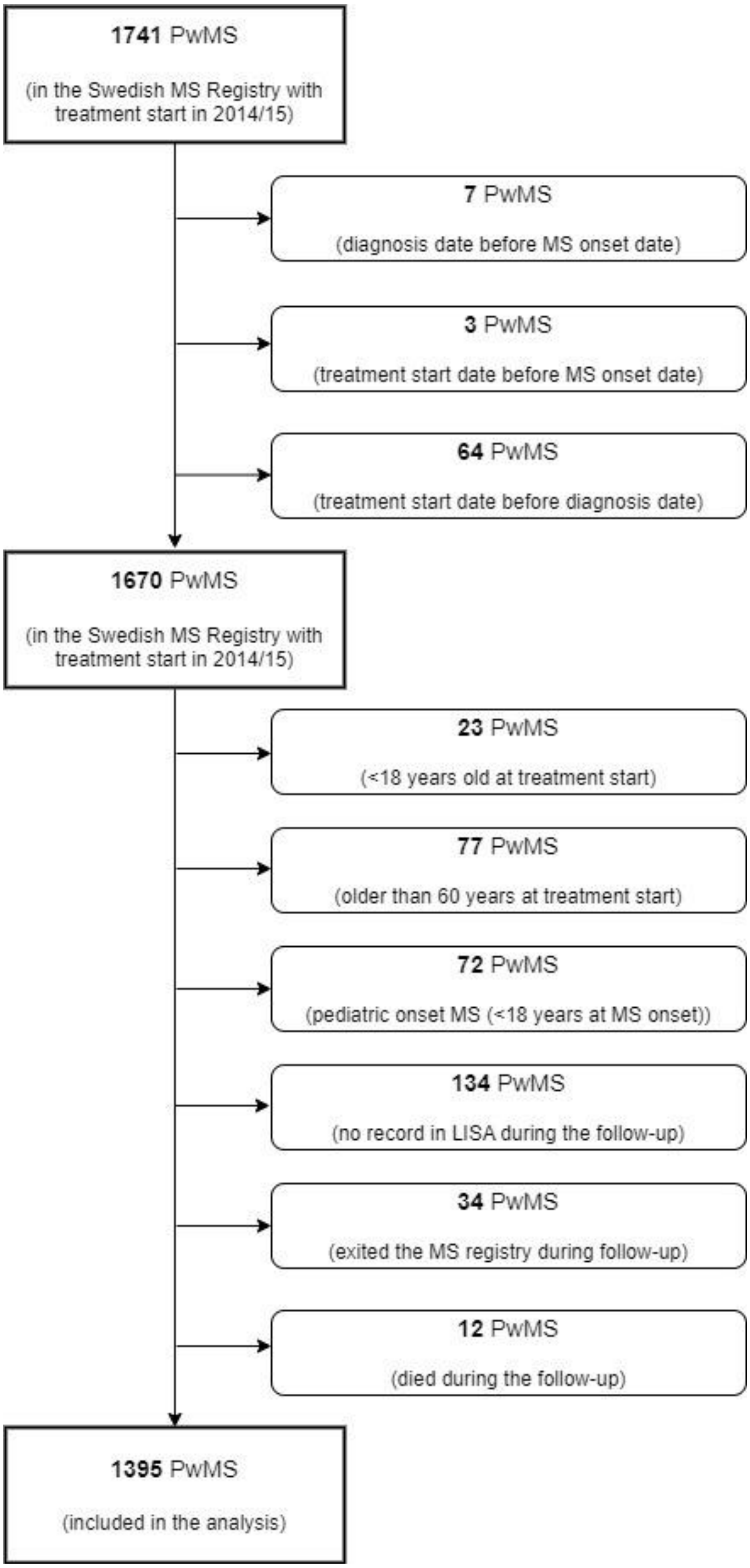

Figure S1. Selection of people with multiple sclerosis included in the study [LISA: Longitudinal Integrated Database for Health Insurance and Labor Market Studies; PwMS: people with multiple sclerosis; MS: multiple sclerosis] [An individual might fulfil more than one exclusion criteria and the number of PwMS could appear more]

Table S2. Quality assessment metrics for the clusters of disease-modifying therapy use trajectories

Cluster number	PBC	HG	HGSD	ASW[a]	ASWw	CH	R2	CHsq	R2sq	HC
**2**	0.779	0.895	0.894	0.658	0.659	1498.8	0.518	3039.0	0.686	0.063
**3**	0.864	0.977	0.976	0.714	0.714	1333.5	0.657	3965.9	0.851	0.013
**4**	**0.807**	**0.968**	**0.967**	**0.692**	**0.693**	**1433.6**	**0.756**	**4788.2**	**0.912**	**0.020**
**5**	0.751	0.953	0.952	0.655	0.656	1313.4	0.791	4406.6	0.927	0.031
**6**	0.740	0.969	0.968	0.691	0.693	1285.1	0.822	4679.9	0.944	0.020
**7**	0.728	0.973	0.973	0.675	0.677	1188.7	0.837	4392.6	0.950	0.018
**8**	0.697	0.968	0.968	0.668	0.670	1119.0	0.850	4015.9	0.953	0.020
**9**	0.703	0.974	0.973	0.672	0.675	1076.6	0.861	4290.6	0.961	0.017
**10**	0.703	0.976	0.975	0.675	0.678	1019.8	0.869	4290.1	0.965	0.016
**11**	0.698	0.979	0.978	0.680	0.685	978.3	0.876	4251.3	0.968	0.014
**12**	0.690	0.980	0.979	0.672	0.676	939.5	0.882	4040.1	0.970	0.014

PBC: Point Biseral Correlation; **HG**: Hubert's Gamma; **HGSD**: Hubert's Gamma Somer's D; **ASW**: Average Silhouette's Width; **CH:** Calinski-Harabasz Index; $\mathbf{R^2}$: Pseudo R^2; **CHsq**: Calinski-Harabasz Index squared; **R2sq** Pseudo R^2; **HC**: Hubert's C

[a]The ASW was among the main statistical criteria assessed in the study to determine quality of clusters using of its cut off points (ASW: 0.71-1.00: strong structure identified; 0.51-0.70: reasonable structure identified; 0.26-0.50: structure is weak and could be artificial. Try other algorithms; ≤0.25:no structure) [5,6].

Table S3. Descriptive statistics on sociodemographic, clinical, health-related quality of life and sickness absence and disability pension data of PwMS by treatment start year (n=1395)

Variable	Treatment start year		Total	X^2	P-value
	2014	2015			
	% (n)	% (n)	% (n)		
**Sex**					
Women	68.8 (487)	69.6 (478)	69.2 (965)	0.07	0.7929
Men	31.2 (221)	30.4 (209)	30.8 (430)		
**Age**					
19-25	14.8 (105)	9.6 (66)	12.3 (171)		
26-35	27.5 (195)	30.6 (210)	29.0 (405)	10.80	**0.0289**
36-45	27.7 (196)	29.0 (199)	28.3 (395)		
46-55	24.0 (170)	23.1 (159)	23.6 (329)		
56-60	5.9 (42)	7.7 (53)	6.8 (95)		
**Age** (mean (SD))	38.7 (10.7)	39.2 (10.5)	38.9 (10.6)	-	0.359[a]
**Birth country**					
Sweden	88.3 (625)	87.8 (603)	88.0 (1228)		
Outside Sweden	11.7 (83)	12.2 (84)	12.0 (167)	0.04	0.8357
**Family composition**					
Married/cohabitant without children	14.4 (102)	14.1 (97)	14.3 (199)		
Married/cohabitant with children	37.3 (264)	38.4 (264)	37.8 (528)	4.13	0.2478
Single without children	42.2 (299)	38.9 (267)	40.6 (566)		
Single with children	6.1 (43)	8.6 (59)	7.3 (102)		
**Living area**					
Big cities	41.0 (290)	38.9 (267)	39.9 (557)	0.94	0.6248
Medium-sized cities	41.1 (291)	41.5 (285)	41.3 (576)		
Rural areas	17.9 (127)	19.7 (135)	18.8 (262)		
**Educational level**					
0-9 years	11.6 (82)	9.9 (68)	10.8 (150)		
10-12 years	46.5 (329)	45.1 (310)	45.8 (639)	3.73	0.2920
>12 years	41.4 (293)	44.8 (308)	43.1 (601)		
Missing	0.6 (4)	0.1 (1)	0.4 (5)		
**MS Type**[b]					
Relapsing remitting	85.5 (605)	85.7 (589)	85.6 (1194)		
Primary progressive	6.4 (45)	5.7 (39)	6.0 (84)	0.30	0.8625
Secondary progressive	8.2 (58)	8.4 (58)	8.3 (116)		
Missing	0.0 (0)	0.1 (1)	0.1 (1)		
**EDSS score** (closest to treatment start)					
0-2.5	68.2 (483)	65.5 (450)	66.9 (933)		
3-5.5	15.5 (110)	17.6 (121)	16.6 (231)	1.82	0.6100
6-8.5	4.0 (28)	3.5 (24)	3.7 (52)		
Missing	12.3 (87)	13.4 (92)	12.8 (179)		
**Comorbidity index** (treat start year)					
0	9.5 (67)	9.6 (66)	9.5 (133)		
1 to 2	50.6 (358)	48.8 (335)	49.7 (693)	1.25	0.7412
3 to 4	25.1 (178)	27.7 (190)	26.4 (368)		
5+	14.8 (105)	14.0 (96)	14.4 (201)		
**Frequency of DMT switch**					
0	42.9 (304)	51.7 (355)	47.2 (659)		
1	37.1 (263)	34.2 (235)	35.7 (498)	13.73	**0.0082**
2	13.6 (96)	9.6 (66)	11.6 (162)		
3+	5.6 (40)	3.8 (26)	4.7 (66)		
No DMT	0.7 (5)	0.7 (5)	0.7 (10)		
**EQ-5D index** (mean (SD)) (within 2 yrs. before or after treatment start)	0.84 (0.13)	0.83 (0.13)	0.84 (0.13)	-	**0.0489**
**EQ VAS score**	69.2 (21.2)	68.1 (22.4)	68.6 (21.8)	-	0.4134
**Sickness absence/disability pension days**[c]					
SA/DP (ym-2) (mean (SD))	35.6 (92.4)	44.6 (103.0)	40.0 (97.7)	-	**0.0398**
SA/DP (ym-1) (mean (SD))	60.3 (107)	60.7 (109)	60.5 (108.1)	-	0.8311
SA/DP (y0) (mean (SD))	93.2 (132)	95.0 (132)	94.1 (131.6)	-	0.5433

DMT: disease modifying therapy; **EDSS**: expanded disability status scale; **EQ-5D:** EuroQol five-dimension questionnaire; **EQ VAS**: visual analogue scale in the EQ-5D; **MS**: multiple sclerosis; **PwMS**: people with multiple sclerosis; SA/DP: sickness absence; **SD**: standard deviation; [a]independent t-test; [b] missing observations=2 [c]P-values are based on Mann Whitney U test; statistically significant results are shown in **bold**

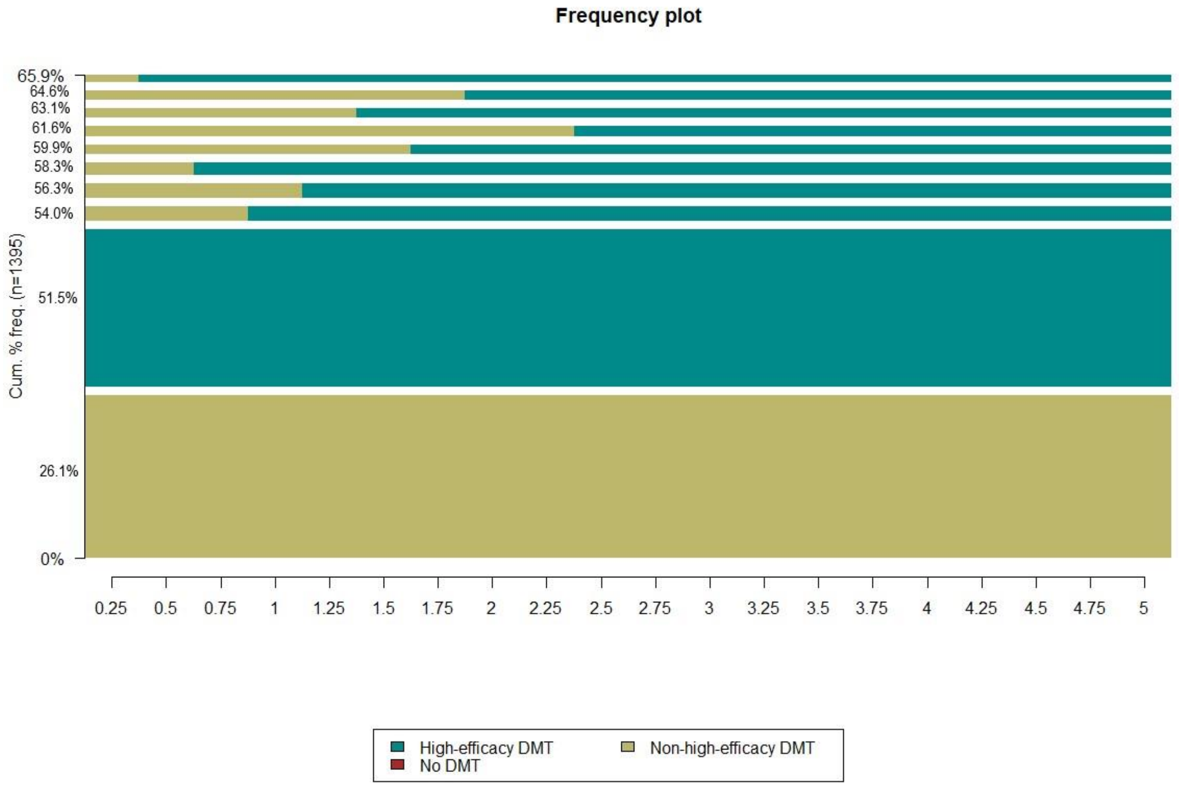

Figure S2. The ten most common sequences of disease-modifying therapies among the people with multiple sclerosis [The sequences of DMT use states shown here present the ten most frequent ones which account for about two-thirds (65.9%) of the total 1395. The total number of sequences (1 for each of the PwMS) are categorized in to the clusters as shown in the findings in the main text of the manuscript based on their similarity/dissimilarity. DMT: disease-modifying therapy]

References

1 Österlund N. MiDAS: Sjukpenning och Rehabiliteringspenning Version 1.02 (in Swedish) [MiDAS: Sickness benefit and Rehabilitation allowance]. 2011.https://www.forsakringskassan.se/wps/wcm/connect/f1e0dce5-e310-4d6d-8076-d4493534c10b/MiDAS_Sjukpenning_och_rehabiliteringspenning_Version_1_02.pdf?MOD=AJPERES (accessed 4 May 2022).

2 Pratt NL, Kerr M, Barratt JD, *et al.* The validity of the Rx-Risk Comorbidity Index using medicines mapped to the Anatomical Therapeutic Chemical (ATC) Classification System. *BMJ Open* 2018;**8**:e021122. doi:10.1136/bmjopen-2017-021122

3 Lu CY, Barratt J, Vitry A, *et al.* Charlson and Rx-Risk comorbidity indices were predictive of mortality in the Australian health care setting. *Journal of Clinical Epidemiology* 2011;**64**:223–8. doi:10.1016/j.jclinepi.2010.02.015

4 Teni FS, Machado A, Murley C, *et al.* Trajectories of disease-modifying therapies and associated sickness absence and disability pension among 1923 people with multiple sclerosis in Sweden. *Multiple Sclerosis and Related Disorders* 2022;**0**. doi:10.1016/j.msard.2022.104456

5 Studer M. WeightedCluster Library Manual: A practical guide to creating typologies of trajectories in the social sciences with R. Published Online First: 2013. doi:10.12682/lives.2296-1658.2013.24

6 Kaufman L, Rousseeuw PJ. *Finding groups in data: an introduction to cluster analysis*. New York: : Wiley 1990.
